# Exogenous Ethylene Promotes Peel Color Transformation by Regulating the Degradation of Chlorophyll and Synthesis of Anthocyanin in Postharvest Mango Fruit

**DOI:** 10.3389/fnut.2022.911542

**Published:** 2022-05-20

**Authors:** Mingmin Chen, Hui Gu, Lirong Wang, Yuanzhi Shao, Rui Li, Wen Li

**Affiliations:** ^1^School of Horticulture, Hainan University, Haikou, China; ^2^Key Laboratory for Quality Regulation of Tropical Horticultural Crops of Hainan Province, Hainan University, Haikou, China; ^3^Key Laboratory of Hainan Province for Postharvest Physiology and Technology of Tropical Horticultural Products, South Subtropical Crops Research Institute, Chinese Academy of Tropical Agricultural Sciences, Zhanjiang, China; ^4^School of Life Sciences, Hainan University, Haikou, China

**Keywords:** ethephon, ripening, color transformation, correlation analysis, mango fruit

## Abstract

Due to geographical location and climatic factors, postharvest storage and preservation of tropical fruits and vegetables are still facing huge challenges. Ethephon (ETH) is widely used as an ethylene donor to achieve the commercial color and flavor of climacteric fruits. However, the effect of ETH on fruit coloration was affected by many factors, such as fruit species, plant hormones, and storage conditions. In this study, the main mango variety “Guifei” in Hainan, China, was used to study the effects of different concentrations of ETH on fruit ripening and coloration during storage at 25°C. Results showed that postharvest treatment with ETH (300, 500, and 900 mg·L^−1^) enhanced the activities of ACS and ACO, stimulated the release of endogenous ethylene, and accelerated fruit softening and color transformation. Compared with control, ETH treatment not only accelerated the breakdown of chlorophyll with higher activities of Chlase and MDCase but also induced the synthesis of carotenoid and anthocyanin with higher activities of PAL, CHI, DFR, and UFGT. Moreover, the changes in DFR and UFGT activities coincided with the increase in ETH concentration. Further, correlation analysis showed that the production of endogenous ethylene induced by ETH was significantly negatively correlated with firmness and chlorophyll content, whereas positively correlated with MDA content and anthocyanin content. This study suggests that the positive effect of ETH on “Guifei” mango color transformation is concentration-dependent within a certain concentration range. Anthocyanin is the main pigment for the red formation of “Guifei” mango, and DFR and UFGT may play critical roles in anthocyanin synthesis. ETH promoted the red coloration by promoting the release of endogenous ethylene and enhancing the activities of anthocyanin synthesis enzymes.

## Introduction

Ethylene is a natural plant hormone that is essential for every stage of fruit ripening. A certain amount of ethylene can promote various attributes of fruits, such as color, texture, and nutritional quality (1–3). Mango (*Mangifera indica*) is very popular because of its pleasure color, characteristic aroma, delicate flavor, and rich nutrition (4). As a typical climacteric fruit, mangoes are usually picked at the physiological maturity period and treated with ethylene to achieve commercial color and flavor (5).

Ethephon [(2-chloroethyl)phosphonic acid, ETH] serves as one of the artificial analogs of ethylene and the role of ETH in fruit ripening has been reported in extensive literature (6). Previous studies have shown that the application of ETH accelerates the ripening process of tomato (7), blueberry (8), pear (9), fig (10), and mango (11). It is well-known that 1-aminocyclopropane-1-carboxylate synthase (ACS) and 1-aminocyclopropane-1-carboxylate oxidase (ACO) are two critical enzymes involved in the biosynthesis of ethylene (12, 13). Previous studies have confirmed that exogenous ethylene treatment increases the production of ethylene and the activity of ACO, whereas treatment with 1-MCP (1-methylcyclopropene, an efficient ethylene antagonist) decreases both (14, 15).

Fruit color is an important trait contributing to fruit quality and market value. Mango fruits are generally classified into green, yellow, and red types based on peel color. Carotenoids and anthocyanins are the important pigments responsible for the yellow or red color of fruits (16). Chlorophyll is one of the photosynthetic pigments, which is widely distributed in mango fruit and masks the contribution of carotenoids and anthocyanins, causing green color retention (17, 18). Previous studies on apples and pears have found that ethylene is involved in the degradation of chlorophyll by regulating the binding between EIN3 and PAO, NYE1, and NYC1 (19–21). Postharvest treatment with ETH increased the color index and chroma and decreased the content of chlorophylls (22). The application of 1-MCP not only significantly inhibits the production of ethylene but also delays the degradation of chlorophyll by increasing the activities of chlorophyllase (Chlase) and Mg-dechelatase (MDCase) (23, 24).

Anthocyanin is another photosynthetic pigment responsible for the color of fruits (17). The biosynthesis of anthocyanin is regulated by a series of enzymes, such as L-phenylalanine ammonia-lyase (PAL), chalcone isomerase (CHI), dihydroflavonol 4-reductase (DFR), and UDP-glucose: flavonoid 3-O-glucosyl transferase (UFGT) (25). For example, the accumulation of anthocyanin coincides with the induction of PAL and UFGT activities (26). Moreover, previous studies on grapes showed that ETH treatment induced the production of endogenous ethylene and hastened the anthocyanin accumulation by stimulating the expression of related genes, such as PAL, CHI, and UFGT (27, 28). However, the application of ETH in strawberries at the large green developmental stage inhibited anthocyanin biosynthesis by downregulated expression of related genes (29).

In addition, the biosynthesis of anthocyanin is a complex biochemical process involving multiple signaling pathways, which is modulated by multiple exogenous agents (30–32). For instance, methyl jasmonate (MeJA)-treated strawberries showed a higher redness of fruit skin and anthocyanin content with an upregulation of *FaUFGT* and *FaANS* genes (30). Exogenous arginine treatment can inhibit the ripening and coloration of postharvest strawberry fruit (33). Some other plant hormones, such as abscisic acid and auxin, are involved in the process of fruit coloration (31, 34). Moreover, elevated CO_2_ treatment participates in fruit color transformation by promoting chlorophyll degradation and flavonoid synthesis (35). These findings indicate that the effect of ETH on fruit color formation was closely associated with fruit species, maturity degree, plant hormones, and storage conditions.

The objective of this study is to investigate the effects of exogenous ethylene at different concentrations on ripening progress and color transformation in “Guifei” mangoes at 25°C. Changes in firmness, MDA content, release of endogenous ethylene, activities of ACS and ACO, peel color change, enzyme activities, and gene expression related to pigment metabolism in stored “Guifei” mangoes were monitored. Correlation analysis was carried out to further analyze the internal complicated relations among exogenous ethylene, endogenous ethylene, ripening process, and color transformation.

## Materials and Methods

### Fruit Materials

All mango (*Mangifera indica* L. cv. Guifei) fruits used in this study were picked up at the mature green stage from a commercial orchard located in Baise City, Guangxi province of China. To avoid mechanical injury, harvested mango fruits were packed in plastic crates, wherein the fruit of each layer was separated with soft fabric, and air-transported to the postharvest laboratory with air conditioning at 25°C and 75–80% relative humidity (RH). Only well-formed fruits with uniform maturity, size, and color were soaked with 0.1% sodium hypochlorite, dried at room temperature, and used for further experiments.

### ETH Treatment

All mango fruits were divided into four groups, and each group consisted of 130 mangoes. The four groups were immersed in 0 (water as control), 300, 500, and 900 mg·L^−1^ ETH solution for 10 min, respectively. After that, every fruit was air-dried at room temperature, packed using a polyethylene bag, and stored in a climatic cabinet (20°C, RH 85%). Then, each group was divided into two categories: one category (10 fruits) was used for monitoring the color change of mango fruit on 0, 3, 6, 9, 12, and 15 days after treatment. The other category (about 120 fruits) was used for the measurement of related physiological parameters.

Mango flesh and peel were separated at each sampling time point. Subsequently, each fresh sample was divided into two parts: one part was used for measurement of the accumulation of MDA and pigments; the other part was frozen in liquid nitrogen, ground into powder, and kept at −80°C to further measure the activities of ACS, ACO, and pigment metabolizing enzymes. Each treatment comprised three replicates and each replicate comprised nine mangoes.

### Determination of Ripening-Related Parameters

#### Fruit Firmness

Fruit firmness was measured using a handheld penetrometer (TA touch 10020, China) equipped with a 5-mm diameter probe. Two opposite points on the equator axis of each peeled fruit were monitored and firmness was expressed as force in newtons (N).

#### Malondialdehyde (MDA) Content

MDA content was determined based on the protocol reported by Lin et al. (36) with minor modifications. The 0.5 g of fresh flesh were mixed with 6 mL of 0.05 M (pH = 7.8) phosphate buffer, rapidly ground to a homogenate on ice, and centrifuged at 12,000 × *g* for 30 min at 4°C. The supernatant (2 mL) was added to 3 mL of 10% TCA with 0.5% thiobarbituric acid, the mixture was rapidly put into a boiling water bath for 10 min, and then centrifuged at 12,000 × *g* for 30 min. The absorbances of the supernatant at 532 and 600 nm were used for MDA content.

#### Production of Endogenous Ethylene

Two mango fruits were put in a 10-L sealed jar for 2 h at 20°C to collect the released ethylene. A 1-mL gas mixture was extracted to test the production of ethylene with a gas chromatograph (GC) system (Agilent 5181-1267, Palo Alto, CA, USA) equipped with TG-5MS column (30.0 m × 0.32 mm × 0.25 μm) and a hydrogen ion flame detector. Each treatment comprised three replicates, and each replicate was sampled and measured three times.

#### Activities of ACS and ACO

The activity of ACS was measured according to the methods in Liu et al. (37) with some modifications. The 0.5 g flesh powder were homogenized with 10 mL of 0.4 M K_2_HPO_4_/KH_2_PO_4_ buffer (pH = 8.5), containing 10 μM pyridoxal-5-phosphate (P-5-P), 5 mM dithiothreitol (DTT), 1 mM Na_2_-EDTA, and 5% (m·v^−1^) polyvinylpyrrolidone (PVP) at 4°C. The mixture was centrifuged at 9,000 × *g* at 4°C for 15 min. After that, 1 mL supernatant was mixed with 1.5 mL of 50 mM HEPES-KOH buffer (pH = 8.5), containing 10 μM P-5-P, 0.25 mM S-adenosyl methionine (SAM), and 1% Triton X-100. The mixture was incubated in a 10-mL sealed test tube at 30°C for 30 min. Finally, 1 mL gas was withdrawn from the headspace to analyze the level of ethylene by GC. One unit (U) of ACS activity was defined as the amount of enzyme that produced 1 nM C_2_H_4_ g^−1^ fresh weight min^−1^.

ACO activity was measured according to the methods in Zhang et al. (38) with some modifications. The 0.5 g flesh powder were homogenized with 6 mL of 0.1 M Tris-HCl (pH = 7.5), containing 10% (m·v^−1^) glycerin, 5% (m·v^−1^) PVP, 5 mM DTT, 0.1 mM FeSO_4_, and 30 mM sodium ascorbic acid. The mixture was centrifuged at 9,000 × *g* for 15 min at 4°C. Afterwards, 1 mL supernatant was mixed with 3 mL of 0.1 M Tris-HCl (pH = 7.5), containing 30 mM NaHCO_3_, 10% (m·v^−1^) glycerin, 0.1 mM FeSO_4_, 1 mM ACC, and 30 mM sodium ascorbic acid. The reaction solution was incubated at 30°C for 1 h, and then 1 mL gas was collected from the headspace for measuring the production of ethylene by GC. One unit (U) of ACO activity was defined as the amount of enzyme that produced 1 nM C_2_H_4_ g^−1^ fresh weight min^−1^. Activities of ACS and ACO were expressed as U·g^−1^·min^−1^.

### Determination of Indicators Related to Fruit Color

#### Measurement of Fruit Color

Fruit color was measured at three evenly distributed equatorial sites of every fruit. L^*^, a^*^, and b^*^ at each site were recorded with a chromameter (CM-700d, Konica Minolta, Japan). L^*^ represents the degree of lightness and its values generally stay within a range from 0 to 100; a^*^ represents redness (+) and greenness (-) on the positive and negative axes; b^*^ represents yellowness (+) and blueness (-) on the positive and negative axes. Each time point comprised six mangoes.

#### Contents of Chlorophyll, Carotenoid, and Anthocyanin

The contents of chlorophyll and carotenoid were measured with an ultraviolet spectrophotometer (UV752N, YoKe Instrument Co., Ltd, Shanghai, China) according to the methods in Xie et al. (39) with minor modifications. The 0.5 g fresh peel was mixed with 80% acetone and extracted three times. The mixture was centrifuged at 12,000 × *g* for 30 min. After centrifugation, the pigment solution was mixed with 80% acetone and brought to a final volume of 10 mL. Contents of chlorophyll and carotenoid were calculated according to the absorbances at 470, 645, and 663 nm.

Anthocyanin content was determined based on the methods reported by Zhang et al. (40) with some modifications. The 0.5 g fresh peel was mixed with 10 mL of 1% cool hydrochloric acid-methanol solution in a test tube with a plug. The mixture was kept away from light at room temperature for 24 h until the peel turned white. After that, a 3-mL reaction solution was used for the assay of anthocyanin content based on its absorbances at 530 and 600 nm; 1% hydrochloric acid-methanol solution was used as the blank control.

#### Activities of Chlase and MDCase

The crude enzyme solution of Chlase and MDCase was extracted according to the methods described by Fukasawa et al. (41) with minor modification. The 0.2 g of peel powder were mixed with 6 mL of 0.1 M phosphate buffer (pH = 6.0), containing 0.2% (v·v^−1^) TritonX-100, 30 g·L^−1^ PVP, 1 mM phenylmethylsulphonyl fluoride (PMSF), and 5 mM L-cysteine. The mixture was homogenized on ice and centrifuged at 12,000 × *g* for 20 min at 4°C. Activities of Chlase and MDCase were assayed based on the absorbance at 667 and 692 nm, respectively.

#### Activities of PAL, CHI, DRF, and UFGT

For PAL and CHI, 0.5 g peel powder was mixed with 5 mL of 0.05 M Na_2_HPO_4_/KH_2_PO_4_ (pH = 7.0), containing 0.05 M ascorbic acid and 0.018 M β-mercaptoethanol. For DFR and UFGT, 0.5 g of peel powder were homogenized with 0.1 M Tris-HCl (pH = 7.5), containing 0.01 M ascorbic acid, 0.005 M dithiothreitol, 0.1% β-mercaptoethanol, and 0.01 M PMSF. The mixture was centrifuged at 12,000 × *g* for 20 min at 4°C. After centrifugation, the enzyme extract was concentrated with ammonium sulfate. Activities of the four enzymes were assayed following the methods described in Chen et al. (42).

### Measurement of Gene Expression

#### Total RNA Extraction and cDNA Synthesis

Total RNA extraction was done with the cetyltrimethylammonium bromide (CTAB) method according to our previous reports (43). The integrity and quality of RNA were verified by 1.5% agarose gel electrophoresis and the A260/A280 ratio. The synthesis of cDNA was carried out using a PrimeScript™ RT reagent Kit with gDNA Eraser (HiScript®, Nanjing, China).

#### Real-Time Quantitative PCR (RT-QPCR)

The RT-qPCR was performed with the SYBR Premix Ex Taq (HiScript®, Nanjing, China) according to its manufacturer's instructions. The primers were designed using the Primer explorer v5 online website (https://primerexplorer.jp/e/), and the primers used in this study are listed in Supplementary Table 1. The qTOWER^3^ G Real-Time PCR System (Wacker Biotech GmbH, Germany) was used for RT-qPCR. The thermal cycling protocol was as follows: 95°C for 5 min, followed by 40 cycles of 95°C for 5 s and 60°C for 30 s. The relative expression was calculated using the 2^−ΔΔCt^ method (44). Each sample has three biological replicates and three technical replicates.

### Statistical Analysis

All data were presented as mean ± standard error (SE). Duncan's multiple range test was used for statistical evaluation using SPSS statistics 20.0 (IBM Corp., USA) with differences being considered significant (*P* < 0.05). Correlation analysis was carried out *via* OriginPro 2021 (Origin Lab, USA) with differences being considered significant (^*^P < 0.05, ^**^P < 0.01, ^***^P < 0.001).

## Results

### Effects of ETH Treatment on Peel Color Transformation of Postharvest Mango Fruit

**Figure 1:**
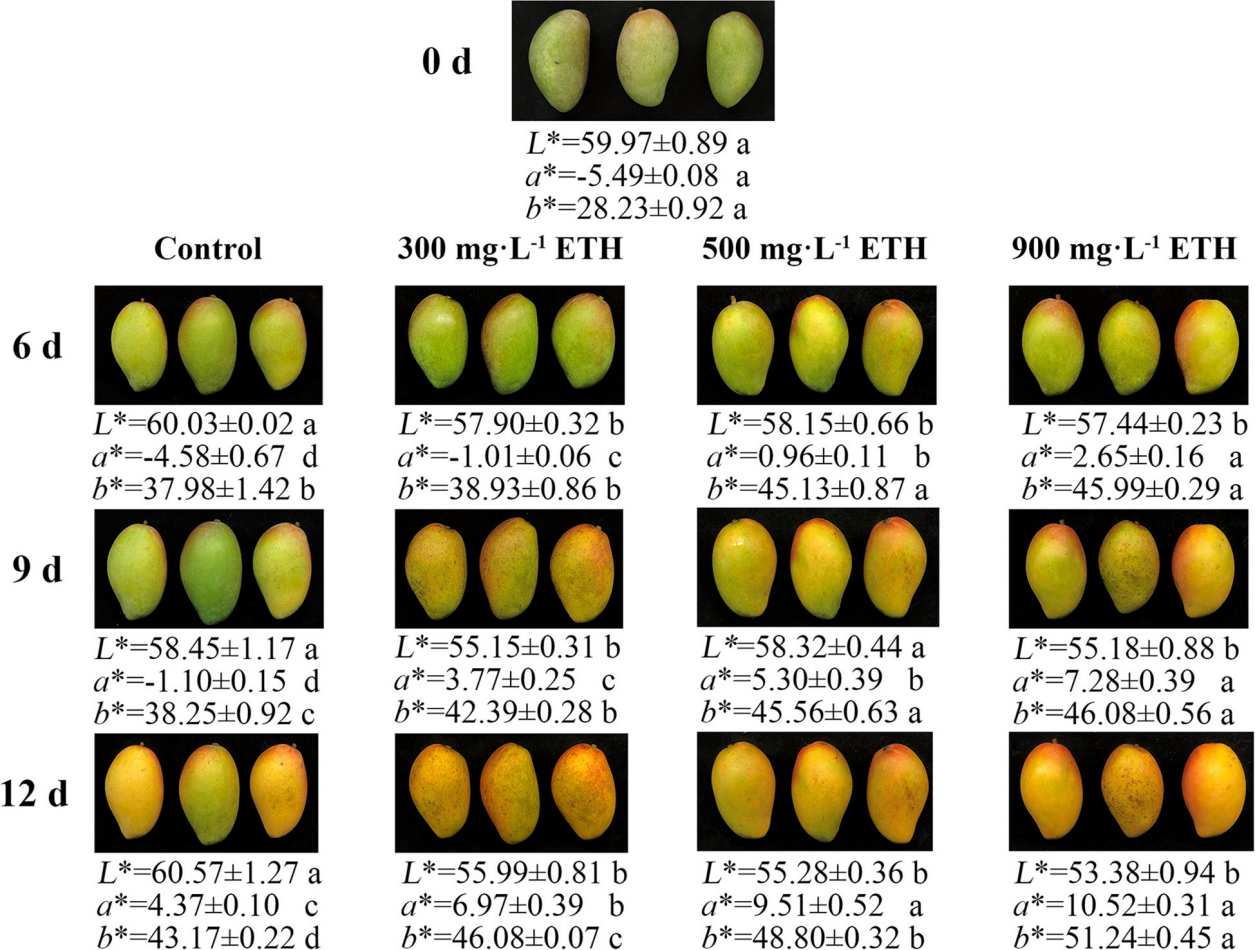
Effects of ETH treatment on peel color transformation in postharvest mango fruit. Photos were taken on 0, 6, 9, and 12 days after treatment.

Before treatment, mango fruit in four groups showed no significant difference in peel color (Figure 1). After treatment, the peel color of control and ETH-treated fruit all began to change from green to red. However, compared with control, the peel color of ETH-treated mango fruit changed earlier and faster. The values of a^*^ and b^*^ in ETH-treated groups were significantly higher than those of the control at 6, 9, and 12 days (Figure 1). Moreover, the speed of peel color change was positively correlated with the application concentration of ETH. As shown in Figure 1, 900 mg·L^−1^ ETH treatment induced the earliest and most pronounced color transformation of mango fruit, followed by the 500 mg·L^−1^ ETH-treated group, and the last was 300 mg·L^−1^ ETH-treated group.

### Effects of ETH Treatment on Ripening Process of Postharvest Mango Fruit

**Figure 2:**
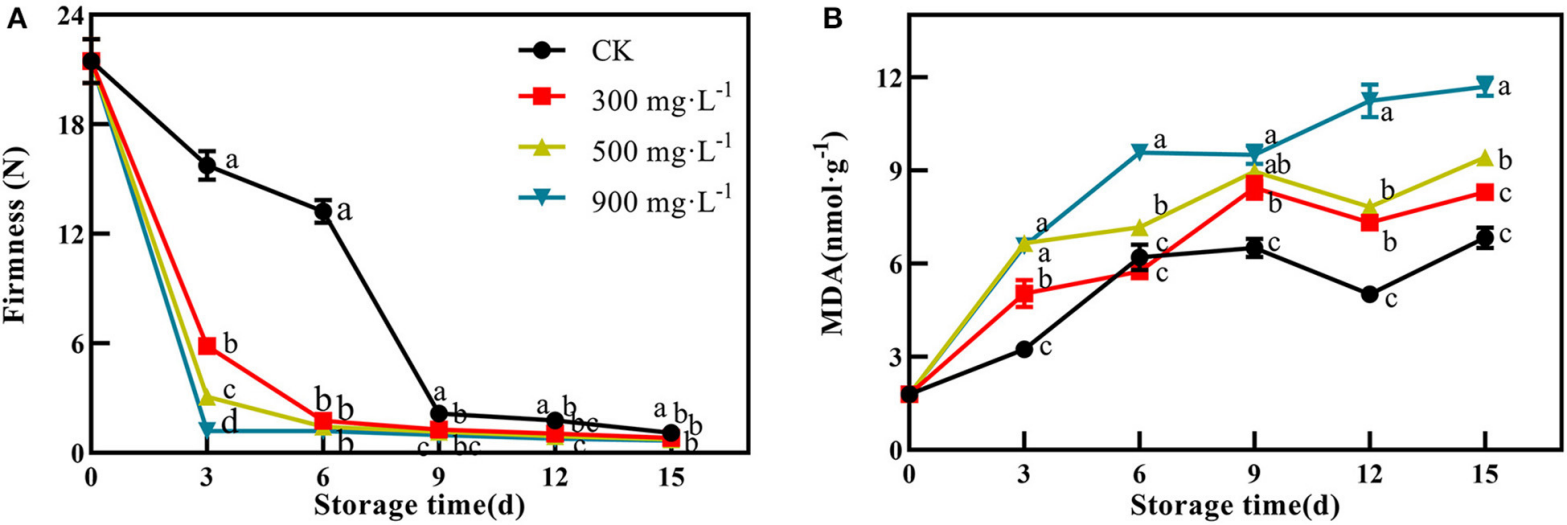
Effects of ETH treatment on firmness **(A)** and MDA content **(B)**. Values followed by different letters are significantly different based on Duncan's multiple range tests at *P* < 0.05. The error bars indicate the standard deviations of three biological replicates.

In this study, firmness and MDA content were used as two important indicators of fruit ripening. After treatment, the firmness of the control fruit were 15.74 N and 13.23 N at 3 and 6 days, respectively (Figure 2A). However, the three ETH-treated groups showed significantly lower firmness than that of the control at 3 and 6 days (Figure 2A, *P* < 0.05). Like the results of peel color, the ETH-mediated softening process of mango fruit was positively correlated with its concentration. For example, the firmness of 300 mg·L^−1^ ETH-treated fruit at 3 days was significantly lower than those of 500 and 900 mg·L^−1^ ETH-treated fruit (Figure 2A, *P* < 0.05).

As shown in Figure 2B, MDA content in control and ETH-treated fruit showed similar change trends after treatment. ETH treatment promoted the accumulation of MDA in mango fruit, and the contents of MDA in the three ETH-treated groups were significantly higher than those in the control group. Especially, both 900 and 500 mg·L^−1^ ETH-treated groups exhibited significantly higher contents of MDA compared with control during the whole storage time (*P* < 0.05).

### Effects of ETH Treatment on Release of Endogenous Ethylene in Postharvest Mango Fruit

**Figure 3:**
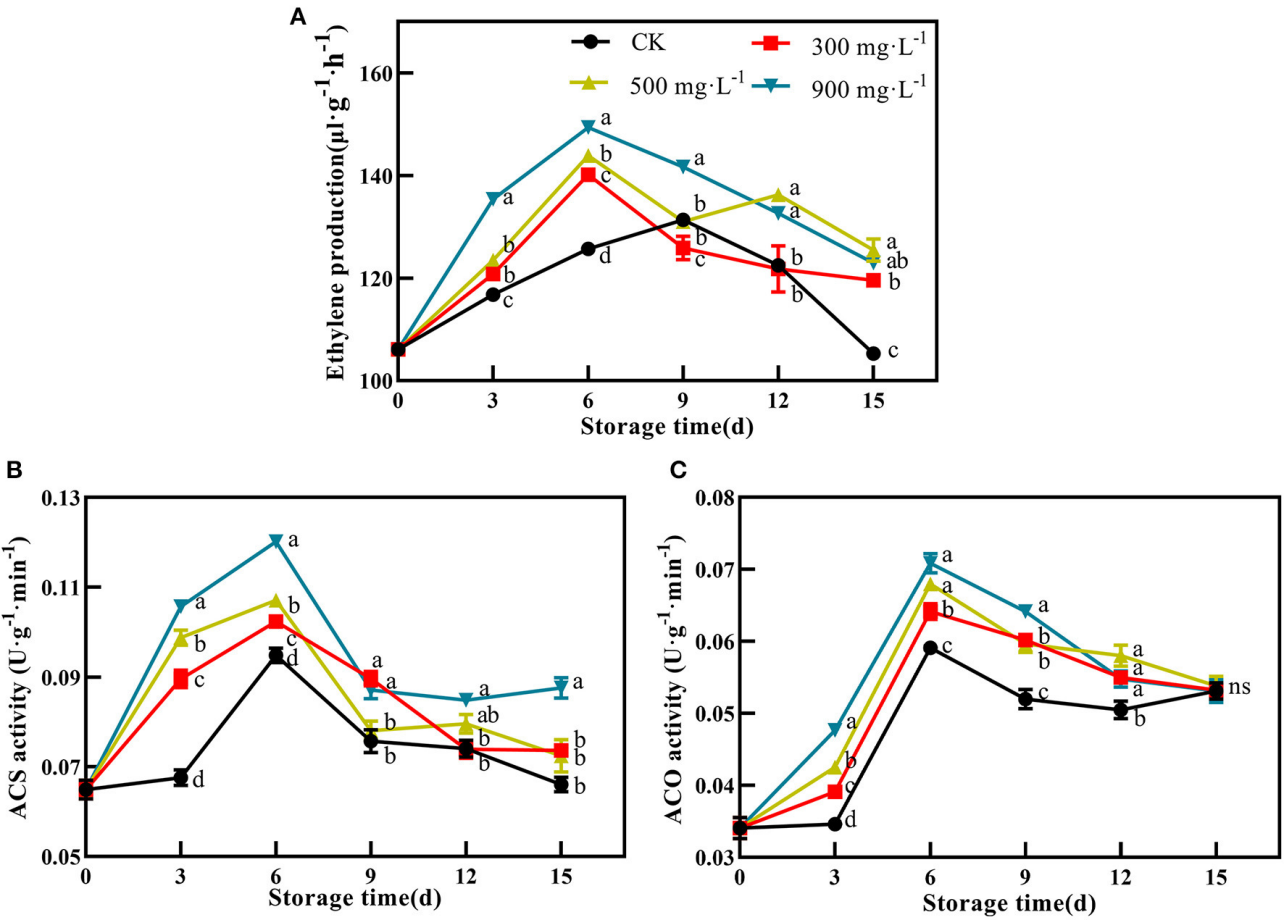
Effects of ETH treatment on production of endogenous ethylene **(A)** and activities of ACS **(B)** and ACO **(C)**. Values followed by different letters are significantly different based on Duncan's multiple range tests at *P* < 0.05. The error bars indicate the standard deviations of three biological replicates.

After treatment, the production of endogenous ethylene in the control fruit peaked at 9 days with 131.36 μL·g^−1^·min^−1^. Compared with control, ETH-treated fruit exhibited earlier and stronger production of endogenous ethylene, which peaked at 6 days with 140.16 μL·g^−1^·min^−1^, 143.87 μL·g^−1^·min^−1^, and 149.41 μL·g^−1^·min^−1^, respectively (Figure 3A, *P* < 0.05). In addition, ETH-induced production of endogenous ethylene was closely related to its application concentration. As shown in Figure 3A, 900 mg·L^−1^ ETH-treated fruit exhibited significantly higher production of endogenous ethylene than the other two ETH-treated groups.

Furthermore, ETH treatment was quite effective in activating the activity of ACS compared with control. After treatment, the activity of ACS in control and ETH-treated fruit increased rapidly, reached a peak at 6 days, and then slowly decreased (Figure 3B). However, ACS activity in ETH-treated groups was significantly higher than that in control at 3 and 6 days (Figure 3B, *P* < 0.05). Similar to ACS, ACO activity of control and ETH-treated groups all peaked at 6 days, but ETH-treated fruit showed significantly higher activities of ACO at 3, 6, 9, and 12 days than control fruit (Figure 3C, *P* < 0.05). The enhanced activities of ACS and ACO contributed to an earlier and stronger accumulation of endogenous ethylene in mango fruit (Figure 3A).

### Effects of ETH Treatment on Contents of Chlorophyll, Carotenoid, and Anthocyanin

**Figure 4:**
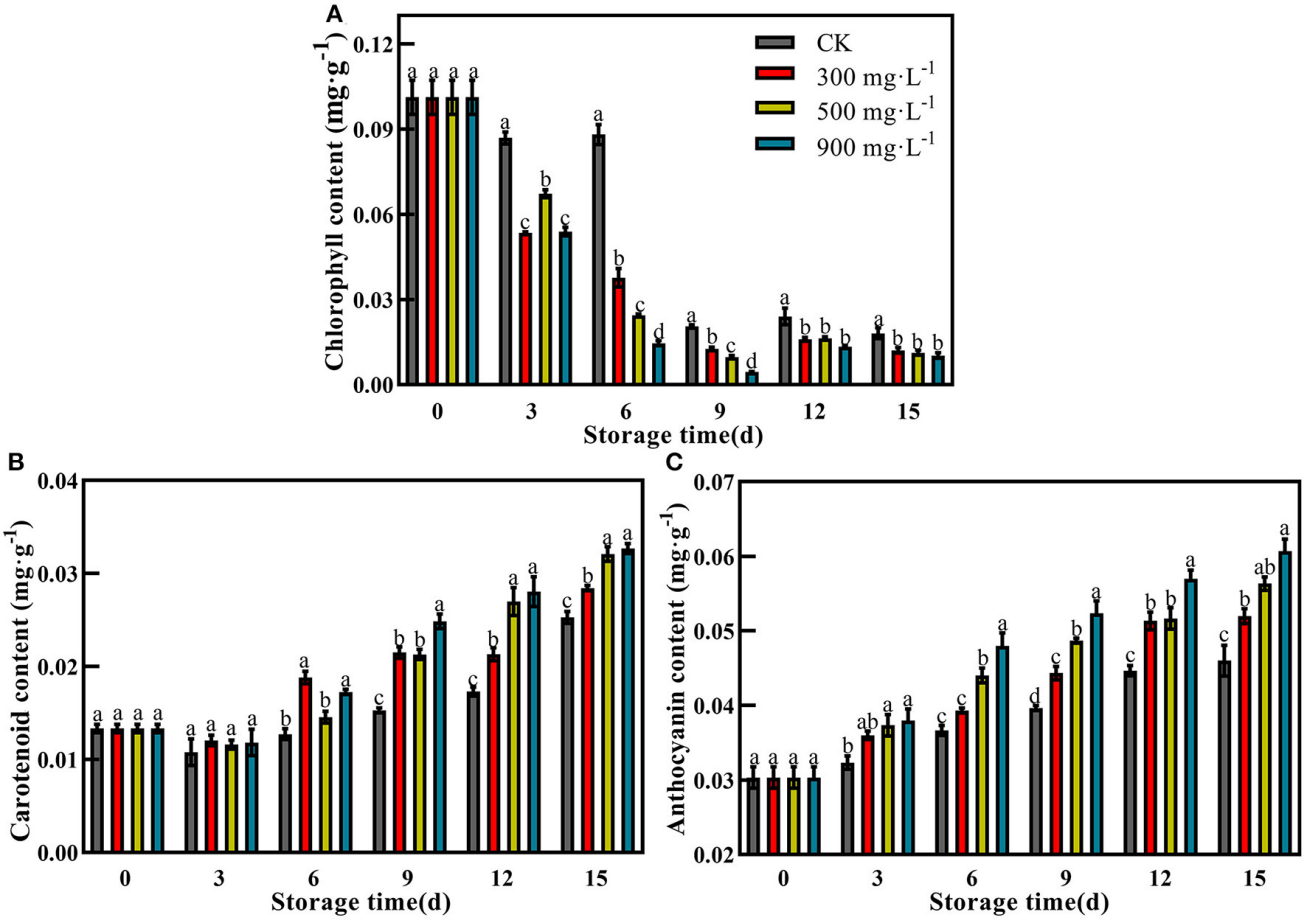
Effects of ETH treatment on contents of chlorophyll **(A)**, carotenoid **(B)**, and anthocyanin **(C)**. Values followed by different letters are significantly different based on Duncan's multiple range tests at *P* < 0.05. The error bars indicate the standard deviations of three biological replicates.

Compared with control, the fruits treated with ETH all showed significantly decreased content of chlorophyll during the whole storage time (Figure 4A, *P* < 0.05). ETH-treated fruit exhibited significantly lower levels of chlorophyll from 3 to 15 days. However, the chlorophyll content of the control fruit showed a relative high level until 6 days. During the mid-storage time, the chlorophyll content of fruit treated with 900 mg·L^−1^ ETH was significantly lower than those of the other two ETH-treated groups. At the end of the storage period, there was no significant difference in chlorophyll content among the three ETH-treated groups (Figure 4A, *P* < 0.05).

In general, the content of carotenoids in the four groups slowly increased after treatment. Carotenoid contents of three ETH-treated groups were significantly higher than that of control from 6 to 15 days (Figure 4B, *P* < 0.05). The highest content of carotenoid was found in fruit treated with 900 mg·L^−1^ ETH at 15 days. Similar to carotenoid, ETH treatment increased the accumulation of anthocyanin in mango fruit (Figure 4C). Moreover, the anthocyanin content of control and ETH-treated fruits showed a significantly increasing trend during the whole storage time (Figure 4C, *P* < 0.05). In addition, 900 mg·L^−1^ ETH-treated fruit exhibited the highest anthocyanin content at 15 days.

### Effects of ETH Treatment on Activities of Pigment Metabolizing Enzymes

**Figure 5:**
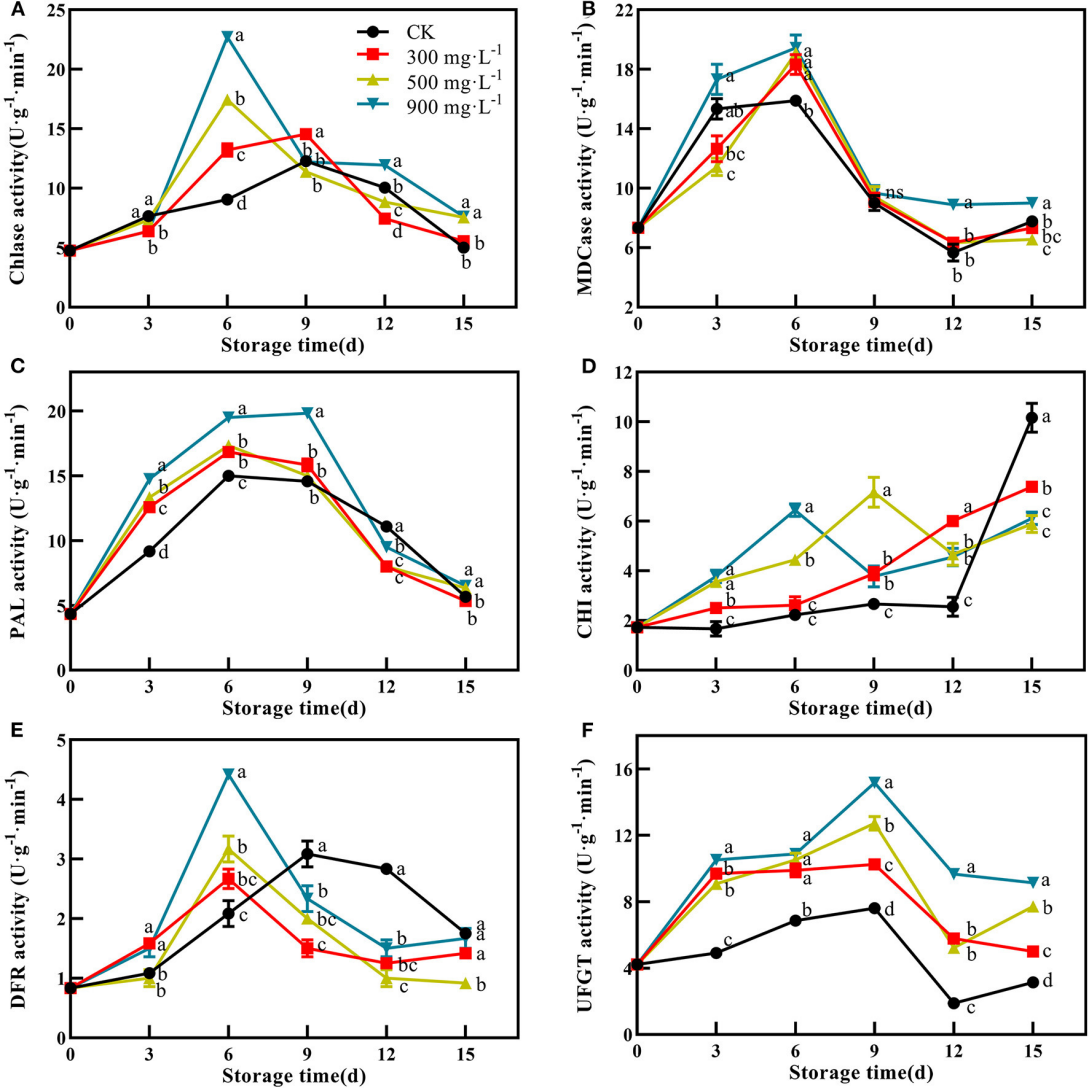
Effects of ETH treatment on activities of Chlase **(A)**, MDCase **(B)**, PAL **(C)**, CHI **(D)**, DFR **(E)**, and UFGT **(F)**. Values followed by different letters are significantly different based on Duncan's multiple range tests at *P* < 0.05. The error bars indicate the standard deviations of three biological replicates.

In this study, control and ETH-treated fruit showed a similar change trend of Chlase activity, which peaked at 6 or 9 days, and then slowly decreased (Figure 5A). The fruit treated with 900 mg·L^−1^ ETH showed the highest activity of Chlase at 6 days with 22.72 U·g^−1^·min^−1^, which was significantly higher than that of the other three groups (Figure 5A, *P* < 0.05). Furthermore, the MDCase activity of each group showed an increasing trend during the first 6 days, followed by a decrease from 6 to 12 days (Figure 5B). At 6 days, the activity of MDCase in ETH-treated fruit was significantly higher than that in control, but there was no significant difference between the three ETH-treated groups (Figure 5B, *P* < 0.05).

PAL, CHI, DFR, and UFGT were four critical enzymes associated with the synthesis of anthocyanin. Compared with control, ETH treatment significantly increased the activity of PAL during the first 9 days, and 900 mg·L^−1^ ETH treatment was most effective in activating the activity of PAL. However, there was no difference in PAL activity between 300 and 500 mg·L^−1^ ETH treatment groups (Figure 5C, *P* < 0.05). As shown in Figure 5D, CHI activity in ETH-treated groups was significantly higher than that in the control group in the early and middle of storage time (*P* < 0.05), but the control fruit exhibited significantly higher activity of CHI than ETH-treated fruit at 15 days (Figure 5D, *P* < 0.05).

Similar to Chlase, the activity of DFR in ETH-treated fruit peaked at 6 days, while the peak of control fruit occurred at 9 days. Moreover, DFR activity in 900 mg·L^−1^ ETH-treated fruit was significantly higher than those in 500 and 300 mg·L^−1^ ETH-treated fruit at 6 days (Figure 5E, *P* < 0.05). From 9 days, the control fruit exhibited significantly higher activity of DFR compared with ETH-treated fruit (Figure 5E, *P* < 0.05). Generally, UFGT activity of the four groups all peaked at 9 days, but UFGT activity in ETH-treated fruit was significantly higher than that in control fruit during the whole storage time (Figure 5F, *P* < 0.05).

### Effects of ETH Treatment on Expression of Genes Involved in Ethylene Biosynthesis and Pigment Mechanism

**Figure 6:**
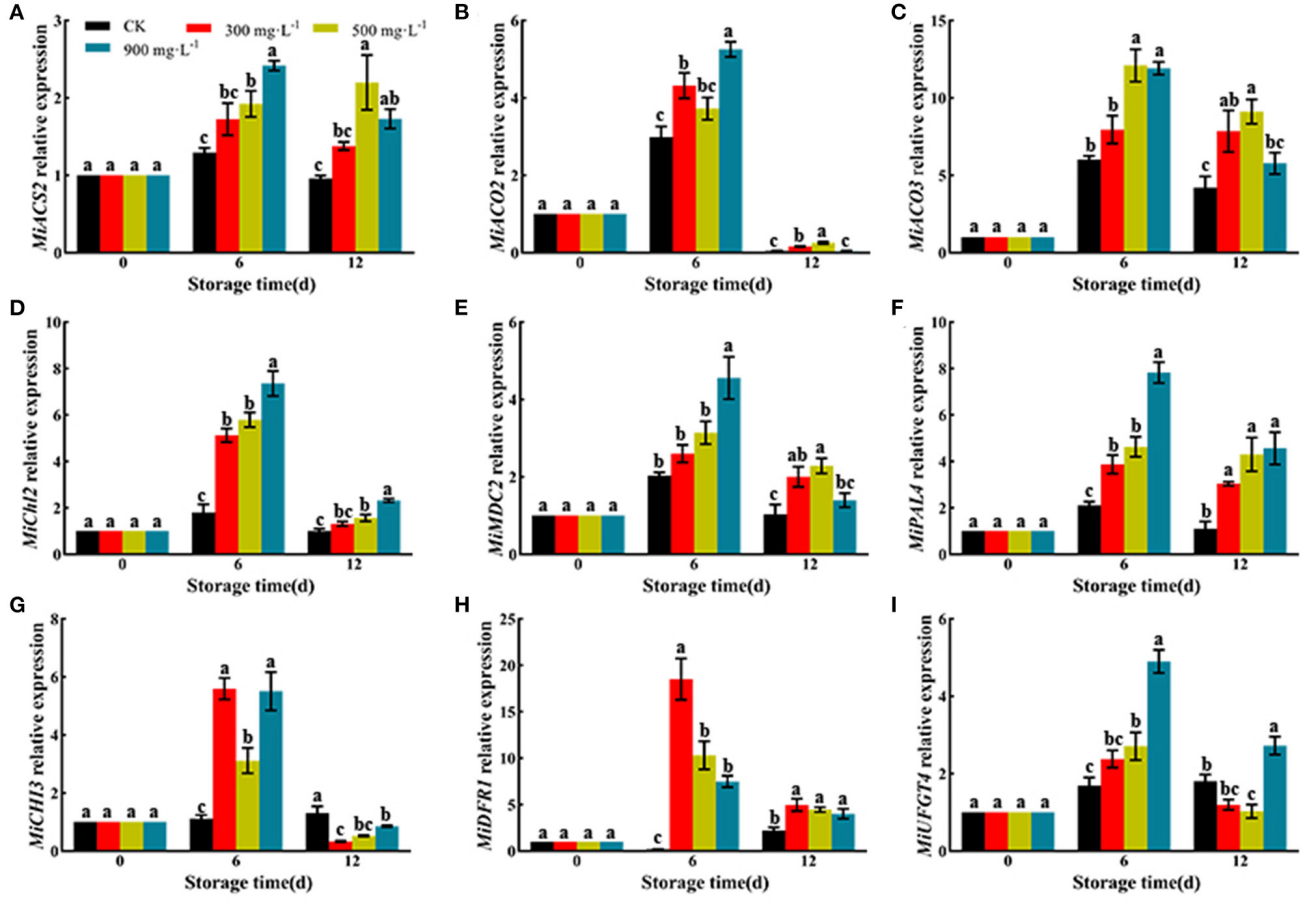
Relative expression of genes involved in ethylene biosynthesis and pigment mechanism including *MiACS2* **(A)**, *MiACO2* **(B)**, *MiACO3* **(C)**, *MiChl2* **(D)**, *MiMDC2* **(E)**, *MiPAL4* **(F)**, *MiCHI3* **(G)**, *MiDFR1* **(H)**, and *MiUFGT4* **(I)**. Values followed by different letters are significantly different based on Duncan's multiple range tests at *P* < 0.05. The error bars indicate the standard deviations of three biological replicates.

As shown in Figure 6, ETH treatment significantly upregulated the relative expression of genes involved in ethylene biosynthesis, such as *MiACS2, MiACO2*, and *MiACO3*. Moreover, ETH treatment significantly upregulated the relative expression of *MiChl2* and *MiMDC2*, which were involved in the degradation of chlorophyll. ETH-treated mango fruit exhibited higher expression of *MiPAL4, MiCHI3, MiDFR1*, and *MiUFGT4*, which contributed to the synthesis of anthocyanin.

### Correlation Analysis Between the Production of Endogenous Ethylene and Fruit Ripening

First, we analyzed the correlation between the production of ethylene and the activities of ACS and ACO. Results showed that the *R*^2^ value between ethylene production and activity of ACS or ACO were 0.89 and 0.85, respectively (Supplementary Figures 1A,B), reflecting a high degree of linear correlation. Second, the *R*^2^ value between ethylene production and firmness or MDA content were 0.84 and 0.85, respectively (Supplementary Figures 1C,D). In addition, the coefficient of the linear equation between ethylene production and firmness was negative (−0.5441), but the coefficient of the linear equation between ethylene production and MDA content was positive (0.1541; Supplementary Figures 1C,D).

### Correlation Analysis Between the Production of Endogenous Ethylene and Parameters Related to Fruit Color

As shown in Table 1, a^*^ and b^*^ were significantly positively correlated with each other, and the Pearson's correlation coefficient (R-value, as follows) between them was 0.71 (*P* < 0.01). Chlorophyll content was negatively correlated with a^*^ and b^*^, and the R values were −0.87 and −0.61, respectively (*P* < 0.05). Moreover, the R-value between the production of endogenous ethylene and chlorophyll content was −0.95 (*P* < 0.001). These results supported that the production of endogenous ethylene was positively correlated with a^*^ and b^*^, and the R values between them were 0.87 and 0.63 (*P* < 0.05), respectively. The R-value between the content of carotenoid and a^*^ was 0.67, but the R-value between the content of anthocyanin and a^*^ was 0.83.

**Table 1:** Correlation analysis between the production of endogenous ethylene and parameters related to fruit color.

	**Ethylene production**	L^*^	a^*^	b^*^	**Chlorophyll content**	**Carotenoid content**	**Anthocyanin content**	**MDCase activity**	**Chlase activity**	**PAL activity**	**CHI activity**	**DFR activity**	**UFGT activity**
Ethylene production	1												
L^*^	−0.49	1											
a^*^	0.865^***^	−0.153	1										
b^*^	0.629^*^	0.225	0.711^**^	1									
Chlorophyll content	−0.948^***^	0.446	−0.865^**^	−0.609^*^	1								
Carotenoid content	0.505	0.092	0.667^**^	0.610^*^	−0.498	1							
Anthocyanin content	0.907^***^	−0.34	0.829^***^	0.754^**^	−0.925^***^	0.393	1						
MDCase activity	0.935^***^	−0.504	0.694^**^	0.535^*^	−0.835^***^	0.345	0.792^***^	1					
Chlase activity	0.854^***^	−0.071	0.918^***^	0.881^***^	−0.862^***^	0.598^*^	0.919^***^	0.739^**^	1				
PAL activity	0.906^***^	−0.564^*^	0.625^*^	0.581^*^	−0.839^***^	0.393	0.835^***^	0.904^***^	0.731^**^	1			
CHI activity	0.845^***^	−0.424	0.852^***^	0.605^*^	−0.872^***^	0.374	0.919^***^	0.673^**^	0.863^***^	0.741^**^	1		
DFR activity	0.894^***^	−0.151	0.908^***^	0.849^***^	−0.881^***^	0.647^**^	0.909^***^	0.789^***^	0.971^***^	0.793^***^	0.850^***^	1	
UFGT activity	0.857^***^	−0.519^*^	0.696^**^	0.489	−0.910^***^	0.252	0.911^***^	0.738^**^	0.713^**^	0.772^***^	0.807^***^	0.729^**^	1

*^a^Chlorophyll, carotenoid and anthocynin represent their respective contents. MDCase, Chlase, PAL, CHI, DFR, and UFGT represent their respective activities. Asterisks in the table represent significant differences as determined by Duncan's multiple range test (*

* *P < 0.05*,

** *P < 0.01,*

*** *P < 0.001)*.

Furthermore, the production of endogenous ethylene was significantly positively correlated with the content of anthocyanin, and the R-value was 0.91 (*P* < 0.001). The R values between anthocyanin content and activities of anthocyanin metabolizing enzymes, including PAL, CHI, DFR, and UFGT were 0.84, 0.92, 0.91, and 0.91 (*P* < 0.001), respectively. Moreover, the production of endogenous ethylene was also significantly positively correlated with the activities of pigment metabolizing enzymes (*P* < 0.001). In addition, the activity of Chlase and MDCase was negatively correlated with chlorophyll content, and the *R*-value was −0.84 and −0.86, respectively (*P* < 0.001).

### Summary of Correlation Analysis of Fruit Ripening and Color Transformation-Related Parameters

**Figure 7:**
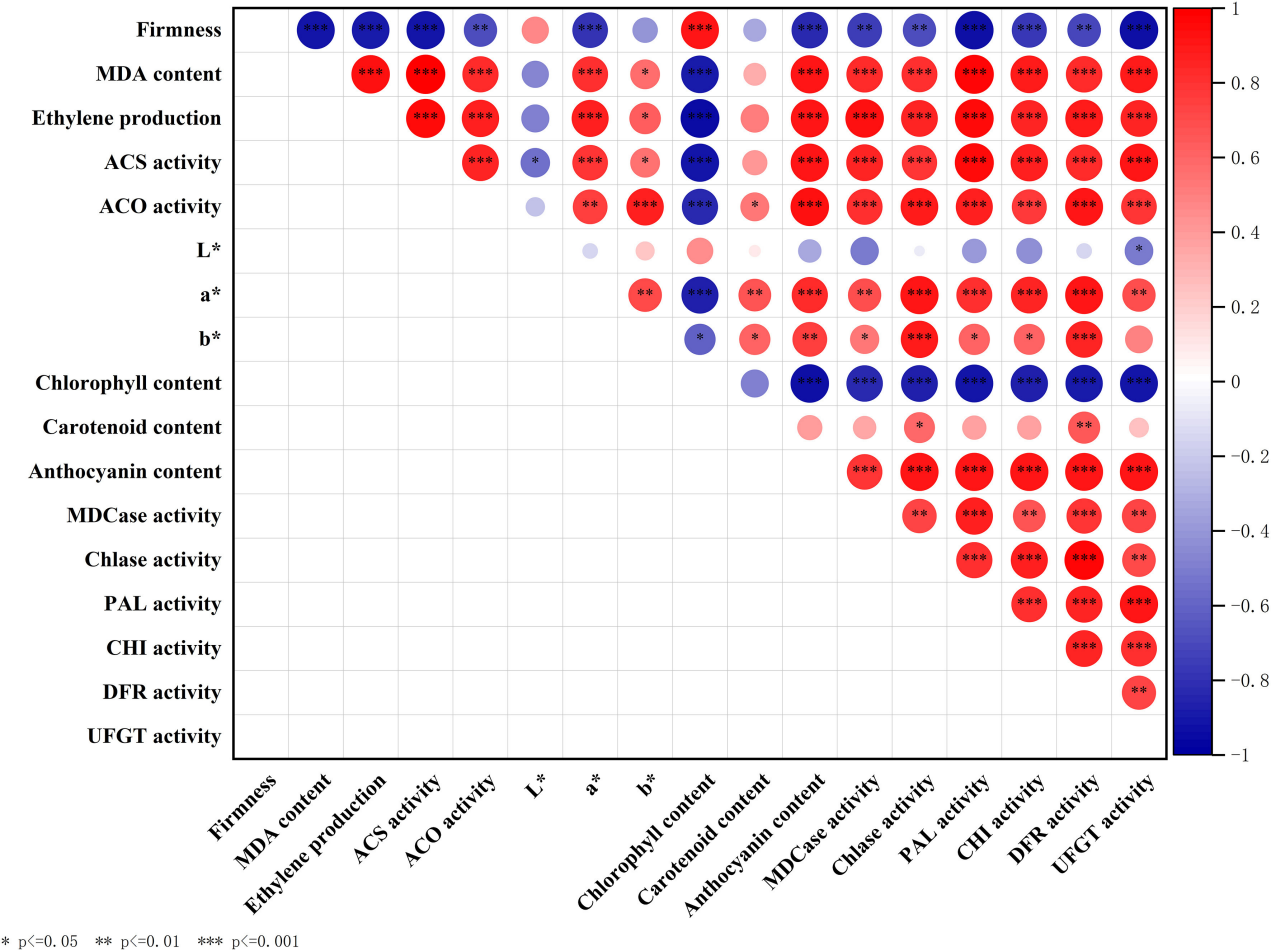
Correlation analysis between parameters including firmness, MDA content, ethylene production, ACS activity, ACO activity, L*, a*, b*, anthocyanin content, chlorophyll content, carotenoid content, Chlase activity, MDCase activity, PAL activity, CHI activity, DFR activity, and UFGT activity. Asterisks indicate significant differences as determined by Duncan's multiple range test (*P < 0.05, **P < 0.01, ***P < 0.001).

We analyzed the inner correlation between all the above parameters involved in fruit ripening and coloring, and the results were shown in Figure 7.

## Discussion

Ethylene can trigger the onset of fruit ripening and play an important role in every period of the ripening process (7). ETH is a kind of ethylene donor, which is widely used in agricultural practice (22). However, it is often found that the peel color of mangoes treated with ETH is normal, but the quality has deteriorated due to excessive maturity (45, 46).

First, firmness and MDA content were selected as two representative indicators to reflect the ripening process of mango fruit (37). During storage, ETH-treated fruit exhibited significantly lower firmness and higher content of MDA than control (Figures 2A,B), which supported the promoting effect of ETH on the ripening process of postharvest mango fruit. Moreover, the advanced emergence of ethylene peak and the increased production of endogenous ethylene indicate that ETH treatment significantly induces the production of endogenous ethylene in postharvest mango fruit (Figure 3A). As shown in Figure 7, the production of endogenous ethylene is significantly negatively correlated with firmness but is positively correlated with MDA content. These results indicate that ETH may accelerate the softening and ripening by regulating the production of endogenous ethylene in postharvest mango fruit (47). Second, previous reports have shown that chlorophyll was the main pigment masking the contribution of carotenoids and anthocyanins, leading to the green color of fruit peel (18). Exogenous ethylene significantly promoted the degradation of chlorophyll in apple (48) and pear (19), and enhanced the synthesis of carotenoid and anthocyanin in papaya (49). In this study, ETH-treated fruit showed significantly lower content of chlorophyll (Figure 4A) and higher contents of carotenoid and anthocyanin (Figures 4B,C), which led to the color transformation of mango fruit (Figure 1).

Moreover, anthocyanins and carotenoids are the most important pigments responsible for the fruit color turning from green to yellow or red or retaining green colors during ripening, and the color parameters at the ripe stage indicated the diversity of fruit colors among the different-colored cultivars (17, 50). For example, there was no significant rise in the content of anthocyanins, but a significant increase in the content of carotenoids during the ripening of yellow-colored mangoes was present (51). In this study, the R-value between chlorophyll content and anthocyanin content was −0.93 (Table 1), while there was no significant correlation between chlorophyll content and carotenoid content (Table 1 and Figure 7). These results implied that compared with carotenoid, the synthesis of anthocyanin contributes more to the peel color transformation of red-colored “Guifei” mangoes. Furthermore, in green mango varieties, the expression of genes involved in the biosynthesis of carotenoids and anthocyanins during the fruit ripening period was low, leading to quite low content of carotenoids and anthocyanins in mango peel, which might be the main reason for its peel color retention (17).

To further explore the inner relationship between the production of endogenous ethylene and fruit color change in “Guifei” mangoes, a correlation analysis was carried out (Figure 7). The highly significant positive correlation between the production of endogenous ethylene and a^*^ (Table 1 and Figure 7) suggested that ETH promoted the color conversion by adjusting the production of endogenous ethylene. Moreover, the production of endogenous ethylene was significantly positively correlated with anthocyanin content but negatively correlated with chlorophyll content, which also supported the critical role of endogenous ethylene in regulating color conversion of postharvest mango fruit. In addition, there is no direct correlation between the production of endogenous ethylene and carotenoid content (Table 1), suggesting that endogenous ethylene was involved in color transformation by regulating anthocyanin synthesis in “Guifei” mangoes.

In addition, color formation is the result of the coordination of multiple hormones and pigment metabolizing enzymes (52). MDCase, Chlase, PAL, CHI, DFR, and UFGT have been regarded as important pigment metabolizing enzymes, participating in the regulation of fruit color formation (18, 25). Compared with control, ETH treatment significantly increases the accumulation of anthocyanin (Figure 4C) by upregulating the expression of *MiPAL4, MiCHI3, MiDFR1*, and *MiUFGT4* (Figure 6) and enhancing the activities of PAL, CHI, DFR, and UFGT (Figure 5). Chlase and MDCase, involved in chlorophyll degradation, are closely related to chlorophyll content in fruit (24), which supports our result that chlorophyll content of mango fruit with higher expression of *MiChl2* and *MiMDC2* and activities of Chlase and MDCase is significantly reduced (Figures 4A, 5A,B). Correlation analysis showed that the production of endogenous ethylene is significantly correlated with activities of MDCase, Chlase, PAL, CHI, DFR, and UFGT (Figure 7), indicating that ETH-induced production of endogenous ethylene regulates mango fruit color transformation by modulating activities of enzymes involved in the degradation of chlorophyll and synthesis of anthocyanin.

Furthermore, previous reports have shown that exogenous ethylene promotes the synthesis of anthocyanin by stimulating the expression of F3H, CHS, LDOX, and UFGT in grape berries (53). Over-expressing of *MdERF1B*, a key ethylene response factor, significantly increases the production of anthocyanins in apples (54). However, previous studies on Chinese pear fruits (55), black rice (25), and *Arabidopsis* (56) have also reported that the accumulation of anthocyanin could be negatively regulated by ethylene signaling components, such as *ETR1* and *EIN3*. Application of Co^2+^, an inhibitor of ethylene biosynthesis, decreased anthocyanin synthesis and PAL activity (57). A similar result was found in cabbage that exogenous ethylene reduced the content of anthocyanin dose-dependently (58). In addition, anthocyanin accumulation in different plant species and organs is regulated by R2R3-MYB and bHLH at the transcriptional level (30). The specific regulatory mechanisms underlying the effect of ethylene on the accumulation of anthocyanin remain to be further studied.

**Figure 8:**
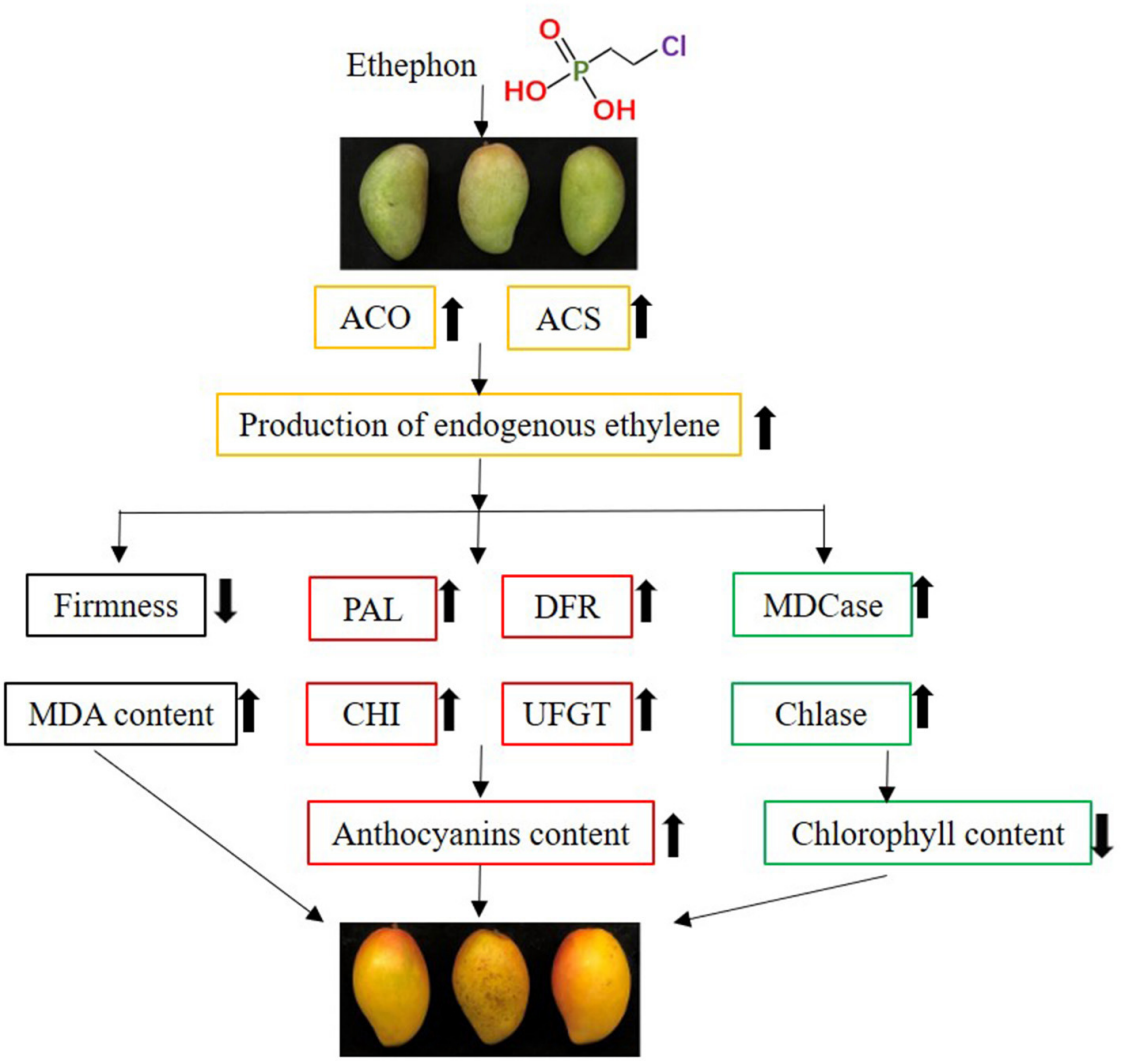
The mode of action of ETH on mango fruit ripening and color transformation.

In this study, we investigated the role of ETH at different concentrations on fruit ripening and color transformation in “Guifei” mangoes during storage at 25°C, and the mode of action of ETH was concluded in Figure 8. Compared with control, postharvest treatment with ETH enhanced the activities of ACS and ACO, stimulated the release of endogenous ethylene, fruit softening, and MDA accumulation, and then accelerated the ripening process of “Guifei” mangoes during storage at 25°C. ETH treatment accelerated the degradation of chlorophyll and the synthesis of anthocyanins and carotenoid in stored mango fruit, accompanied by higher activities of Chlase, MDCase, PAL, CHI, DFR, and UFGT than control. Moreover, the changes in DFR and UFGT activities coincided with the increase in ETH concentration. Further analysis showed that the production of endogenous ethylene highly significantly correlated with activities of the above enzymes, suggesting that ETH might promote mango fruit color transformation by enhancing activities of these pigment metabolizing enzymes. In this study, we analyzed whether and how ETH regulates the ripening process and color transformation of “Guifei” mangoes at the physiological level. These results not only give new perspectives about the role of ETH in mango fruit ripening and color transformation but also provide a theoretical basis for the application of exogenous ethylene in postharvest storage.

## Data Availability Statement

The original contributions presented in the study are included in the article/Supplementary Material, further inquiries can be directed to the corresponding authors.

## Author Contributions

MC, YS, and RL: methodology. MC: software, formal analysis, investigation, and data curation. YS: validation and writing—review and editing. HG and YS: resources. MC and RL: writing—original draft preparation and visualization. RL, YS, and WL: supervision. YS and WL: project administration. RL and WL: funding acquisition. All authors have read and agreed to the published version of the manuscript.

## Funding

This research was funded by the National Natural Science Foundation of China (Grant No. 32072275), the Construction Program of Preservation Technology Association for Tropical Fruits and Vegetables in Hainan Province (Grant No. HDLM201901), and the Start-Up Grant Program in Hainan University (Grant No. KYQD(ZR)-22127).

## Conflict of Interest

The authors declare that the research was conducted in the absence of any commercial or financial relationships that could be construed as a potential conflict of interest.

## Publisher's Note

All claims expressed in this article are solely those of the authors and do not necessarily represent those of their affiliated organizations, or those of the publisher, the editors and the reviewers. Any product that may be evaluated in this article, or claim that may be made by its manufacturer, is not guaranteed or endorsed by the publisher.

## Supplementary Material

Supplementary Figure 1 Linear analysis between the production of endogenous ethylene and parameters involved in fruit ripening.

Supplementary Table 1 Primers were used for RT-qPCR in this study.

## Abbreviations

ETH: 2-chloroethylphosphonic acid
1-MCP: 1-methylcyclopropene
MDA: Malondialdehyde
ACS: 1-aminocyclopropane-1-carboxylate synthase
ACO: 1-aminocyclopropane-1-carboxylate oxidase
Chlase: chlorophyllase
MDCase: Mg-dechelatase
PAL: L-phenylalanine ammonia-lyase
CHI: chalcone isomerase
DFR: dihydroflavonol 4-reductase
UFGT: UDP-glucose: flavonoid 3-O-glucosyl transferase.
