# Supplementary figures and images for: Exogenous Ethylene Promotes Peel Color Transformation by Regulating the Degradation of Chlorophyll and Synthesis of Anthocyanin in Postharvest Mango Fruit
Source: Front Nutr. 2022 May 20;9:911542. doi: 10.3389/fnut.2022.911542 (PMC9165547; doi:10.3389/fnut.2022.911542)

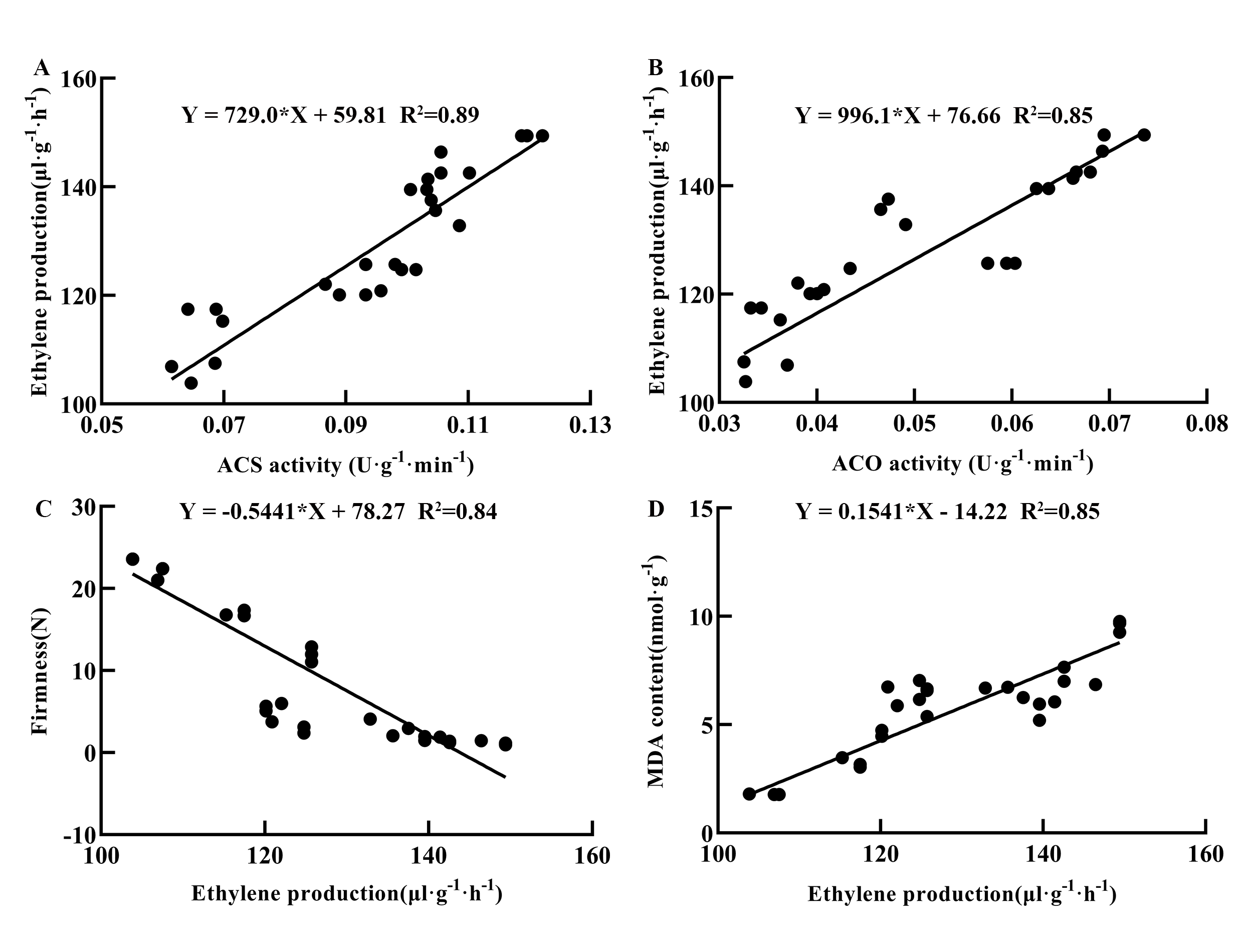

Supplement: Supplementary Figure 1 — Linear analysis between the production of endogenous ethylene and parameters involved in fruit ripening. [file Image_1.TIF]
