# Supplementary material for: Exogenous Ethylene Promotes Peel Color Transformation by Regulating the Degradation of Chlorophyll and Synthesis of Anthocyanin in Postharvest Mango Fruit
Source: Front Nutr. 2022 May 20;9:911542. doi: 10.3389/fnut.2022.911542 (PMC9165547; doi:10.3389/fnut.2022.911542)
Supplement: Supplementary Table 1 — Primers were used for RT-qPCR in this study. [file Table_1.docx]

**Table S1 Primers for RT-qPCR in this study.**

| **Gene** | | **Forward Primer（5'-3'）** | | **Reverse Primer（5'-3'）** |
| --- | --- | --- | --- | --- |
| *MiACS2* | | ACGTTTCACCAGGCTCTTCC | | CGAAGATTCTGCTGCCAACG |
| *MiACO2* | | TGGCGGCCGAATGTCAATAG | | TGGAACTTCAAGCCGGCATA |
| *MiACO3* | | AGGCACTCGGACAATGACTT | | CGCCAGTACTCTATGCTCCA |
| *MiChl2* | | GGATGAGGTTGGTGCTCAGT | | CGCAAGGACCCTGATTATGA |
| *MiMDC2* | | TGAACAAGACAGCAGGAGCA | | GCTTCCACTCTGCCACAACT |
| *MiPAL4* | | TGGCCAAGAAACTCCTGACA | | CTCCTTTTCACCATTGGCCA |
| *MiCHI3* | | CCCCGAGTATTGTGGACCAG | | GCGATCGGAGTGTACGTTGA |
| *MiDFR1* | | TCTTTCATGTCGCCACTCCC | | GGGTTCCTGCAGATGATGTGA |
| *MiUFGT4* | | ACGGCTGGGATTGGAAAAGT | | AGCGTTCAATTGTTGCTCCG |
| *MiACTIN* | | AATGGAACTGGAATGGTCAAGGG | | TGCCAGATCTTCTCCATGTCATCCCA |
|  |  | |  | |
